# Recovery of Flow-Mediated Vasodilatation after Repetitive Measurements Is Involved in Early Vascular Impairment: Comparison with Indices of Vascular Tone

**DOI:** 10.1371/journal.pone.0083977

**Published:** 2014-01-02

**Authors:** Hatsumi Inaba, Kyosuke Takeshita, Yasuhiro Uchida, Motoharu Hayashi, Takahiro Okumura, Akihiro Hirashiki, Daiji Yoshikawa, Hideki Ishii, Koji Yamamoto, Takayuki Nakayama, Masaaki Hirayama, Hiroyuki Matsumoto, Tadashi Matsushita, Toyoaki Murohara

**Affiliations:** 1 Department of Clinical Laboratory, Nagoya University Hospital, Nagoya, Japan; 2 Department of Cardiology, Nagoya University Graduate School of Medicine, Nagoya, Japan; 3 Department of Transfusion, Nagoya University Hospital, Nagoya, Japan; 4 Department of Transfusion, Aichi University Hospital, Nagoya, Japan; University Medical Center Utrecht, The Netherlands

## Abstract

In repetitive measurements of flow-mediated dilatation (FMD), the duration of the interval between measurements remains controversial. In this pilot study, we conducted three sequential measurements of low–flow-mediated constriction (L-FMC), FMD and flow-mediated total dilation (FMTD; L-FMC+ FMD) at baseline and intervals of 15 and 60 min in 30 healthy males. FMD_15_, L-FMC_15_, and FMTD_15_ were significantly lower than the respective first measurements, but all indices showed full recovery at 60 min in all subjects. The baseline diameter was slightly increased at 15 min and restored at 60 min, but the maximum diameter, and the baseline and reactive flow velocity unchanged. We examined the relationship between recovery rate of FMTD at 15 min (FMTD-R) and cardio-ankle vascular index (CAVI). Univariate analysis showed moderate correlation between FMTD-R, and CAVI and L-FMC_0_. Patients were divided according to FMTD-R value; the low-FMTD-R group [below the median value (−26.2%)] included a significantly higher proportion of smokers and higher CAVI values than the high-FMTD-R group. The reproducibility of FMTD and FMTD-R was evaluated in another group of 25 healthy subjects. The range of variation across measurements was 1.1% for FMTD and 4.6% for FMTD-R; with intraclass correlation coefficients of 0.93 and 0.95, respectively. The present study demonstrated blunted recovery of FMD within 15 min, suggesting the need for selection of a more adequate interval between measurements to avoid underestimation of FMD in subsequent measurements. The findings demonstrated the reproducibility of FMTD-R and FMTD measurements, and that FMTD-R might be involved in arterial stiffness and early vascular impairment in the healthy subjects.

## Introduction

The vasodilatory response to shear stress depends on the release of various relaxing factors, e.g., nitric oxide (NO), from endothelial cells. A change in radial artery diameter in response to increased shear stress by reactive hyperemia is recognized as an index of flow-mediated dilation (FMD) in the clinical setting. FMD is an excellent noninvasively measured tool to detect early endothelial dysfunction and low FMD is considered a cardiovascular risk factor [1].

Clinical observation suggests that increased arterial stiffness is another risk factor for cardiovascular disease. Pulse wave velocity (PWV) correlates with vascular elasticity and is used as an index for arterial stiffness. Since there is no correlation between brachial FMD and PWV in healthy subjects [2], [3], the two indices may reflect distinct and independent stages in the complex process of atherosclerosis. Since endothelial cells regulate vascular function in collaboration with smooth muscle cells (SMCs), endothelial dysfunction, induced by atherogenic stimuli, including diabetes, dyslipidemia, and oxidative stress, affects SMC function, resulting in the development of vascular failure [4]. While PWV is affected by changes in instantaneous blood pressure, the cardio-ankle vascular index (CAVI) is a blood pressure-independent index of systemic arterial stiffness, and is often used as a marker of early arteriosclerosis associated with hypertension, diabetes mellitus, dyslipidemia and smoking [5]. There is little or no information on the relationship between FMD and CAVI.

With regard to flow-induced changes in radial arterial diameter, the endothelial response to reduced blood flow by forearm compression is characterized by initial narrowing of the blood vessel. This low–flow-mediated vasoconstriction (L-FMC) reflects the vascular/endothelial response to resting levels of shear stress and provides information on vascular impairment in patients with coronary heart disease [6], [7], [8]. On the other hand, arterial dilatation is observed in response to sudden increase in blood flow. This FMD reflects the capacity of the endothelium to modify the biosynthesis and release of mediators such as NO to produce vasodilation [6]. FMD has shown promising results in cardiovascular risk stratification in the elderly and early vascular impairment in the young population [9], [10]. Previous studies identified endothelial dysfunction in the initial stages of atherosclerosis, long before the development of atherosclerotic lesions or clinical events [11]. Smoking status, which is a major factor in cardiovascular disease, is associated with impairment of endothelium-dependent arterial dilation even in asymptomatic young healthy adults [12]. Since reduced endothelial response is often associated with subclinical changes without obvious pathological evidence of atherosclerosis in young adulthood, assessment of vascular physiology could provide early warning regarding future vascular impairment.

Based on the guidelines for measurement of FMD issued by The International Brachial Artery Reactivity Task Force [13], measurement of FMD requires two longitudinal studies conducted 10- to 15-min apart, with a rest period to reestablish the baseline conditions. However, there is little or no data to confirm the recovery of endothelial function within 10 to 15 min.

In this pilot study, we monitored changes in physiological indices during measurement of FMD at 15 and 60 min intervals. The results showed blunted changes in flow-mediated vascular diameter after 15 min interval. We hypothesized that the reduced recovery of the vascular responses is related to early impairment of endothelial/vascular function. We also compared FMD recovery with FMD-related indices and another index of early vascular impairment, CAVI. We further investigated patient characteristics, including prevalence of smoking, and differences in these vascular physiological indices based on the FMD recovery rate. Finally, we also assessed the reproducibility and repeatability of FMD recovery in a group of healthy subjects. The results suggested that the FMD recovery rate provides information on proper intervals for repeated FMD measurements and underlying impaired vascular function.

## Methods

### Ethics Statement

The experimental procedures and potential risks were explained prior to the study. All participants provided written informed consent to participate. The Institutional Review Board of Nagoya University approved this study and informed consent was obtained from each subject. The study conformed to the principles of the Declaration of Helsinki.

### Participants

The pilot study included 30 healthy men (nonsmokers: 18, current smokers: 12) who participated in a detailed study designed to evaluate FMD-related indices. We defined current smokers as those who had continued smoking (>20 cigarettes per day) for more than 10 year at the time of the study. Each subject underwent medical examination within 3 months of enrolment, and all data were within the normal ranges (Table 1). The study also included another group of 25 healthy males who participated in a study to assess the reproducibility of FMTD-R and FMTD measurements. Each subject underwent carotid artery echography before the FMD and CAVI measurements. All studies were performed around 2:00 PM. Participants were in a fasting state for at least 6 h before the study.

**Table 1:** Clinical characteristics of the patient population divided according to FMTD-R.

	All (n = 30)	Good (n = 15)	Poor (n = 15)
Age (years)	41.0±11	38.0±8.0	42.8±13
Systolic blood pressure (mm Hg)	119.6±9.6	121.0±9.9	119.4±8.8
Diastolic blood pressure (mm Hg)	74.3±8.0	75.9±6.6	73.8±8.7
Body mass index (kg/m^2^)	22.4±2.2	22.6±2.6	22.3±1.7
Heart rate (beats/min)	60.5±7.9	63.3±7.8	57.9±7.3
Total cholesterol (mg/dl)	178±23	167±18	191±21
High-density lipoprotein-cholesterol (mg/dl)	59.5±11	57.4±13	61.5±9.8
Low-density lipoprotein-cholesterol (mg/dl)	102±24	101±15	105±32
Triglyceride (mg/dl)	99.8±36	96.8±40	104.8±34
Glucose (mg/dl)	89.1±8.6	88.9±9.0	89.5±8.9
Arterial diameter at baseline (mm)	4.07±0.48	4.11±0.40	4.04±0.56
Smoker (%)	40	13.3	66.7*

Values are mean ± SD or percentage of patients.

*p<0.004, compared with the good FMTD-R group (Fisher's exact probability test).

### Measurement of brachial artery diameter

FMD was measured according to the international standards [13]. The brachial artery was imaged using an instrument equipped with software for monitoring the brachial artery diameter and blood flow velocity; the system comprised a 7.5-MHz linear array transducer and a novel stereotactic probe-holding device (UNEX EF 18G; Unex Co., Nagoya, Japan) [14]. Continuous recordings of B-mode images and A-mode waves of the brachial artery in the longitudinal plane were obtained. A segment with clear near (media-adventitia) and far (intima-inner lumen) interfaces was manually determined. These border interfaces were identified automatically based on the A-mode waves, and the diastolic diameter of the brachial artery per beat was synchronized with the electrocardiographic R-wave and was tracked automatically. Subjects were rested in the supine position for 30 min in a quiet air-conditioned room (22–24°C). The right brachial artery was scanned in longitudinal sections 1–10 cm above the elbow, the skin surface was marked and the arm was kept in the same position during the study. A pneumatic cuff placed around the forearm was inflated for 5 min to at least 50 mmHg above systolic pressure. The diameter of the brachial artery was scanned and recorded at baseline before cuff inflation, and continuously from the release point to 2 min after cuff deflation to obtain the maximum diameter during reactive hyperemia. The diameter of the artery was measured from one media–adventitia interface to the other at end-diastole, coincident with the R-wave on the continuously recorded electrocardiogram [15]. L-FMC was calculated from the mean arterial diameter during the last 30 s before cuff release and expressed as percentage change from baseline [6]. Traditional FMD was calculated as the maximum percent increase in arterial diameter during continuous measurement of arterial diameter in the 4.5 min following cuff deflation. Finally, flow-mediated total dilation (FMTD) as a composite endpoint was calculated as the sum of the absolute values of FMD and L-FMC [6]. The artery diameter was recorded in the same manner at 15 min interval after the first measurement and at 60-min intervals after the second measurement. All data were analyzed in a randomized, blinded fashion. The FMTD-R was defined as follow: FMTD-R  =  [100× (FMTD_15_ − FMTD_0_)/FMTD_0_]. The subjects were divided into two groups according to FMTD-R (using the median value of −26.2% as the cutoff value); the high and low FMTD-R groups.

### Measurement of CAVI

As reported previously [5], CAVI was measured noninvasively by experienced blinded technicians with a VaSera CAVI instrument (Fukuda Denshi Co., Tokyo) using a standard protocol [16]. The CAVI value is based on the stiffness parameter calculated using the following formula: ln(Ps/Pd) × 2ρ/ΔP × PWV2 (where *ρ*: blood density, *Ps*: SBP, Pd: DBP, *ΔP*: Ps − Pd, *PWV* between the aortic and ankle values) [5]. The subjects rested on a bed in a supine position for 10 min before the measurements.

#### Reproducibility and repeatability analysis

Fifty studies were analyzed twice by two independent observers for reproducibility (variability between observers) analysis. For repeatability (variability between occasions), 25 healthy male subjects (age range 24 to 64 years) underwent measurements of FMTD-R and FMD on two occasions separated by 24 h. The appropriate intraclass correlation coefficient and range of variation (mean of the absolute differences between consecutive measurements) was calculated. Bland-Altman plots were constructed for both FMTD-R and FMD, and the coefficient of interclass repeatability was calculated (1.96 times the standard deviation of the difference between consecutive measurements).

#### Statistical analysis

Data are summarized as mean±SD for quantitative variables. Serial changes in L-FMC, FMD and FMTD between baseline, 15 min and 60 min after the first examination were compared using the Wilcoxon signed-rank test. Based on the nonlinearity of the data, we used the Spearman's correlation coefficients for analysis of L-FMC, FMD, FMTD and CAVI. Values of indices of L-FMC, FMD, FMTD and CAVI were compared between the high and low FMTD-R groups by the Mann-Whitney or Fisher's exact probability test. Statistical significance was defined as p<0.05.

## Results

### Patient characteristics

The study subjects were 30 healthy male subjects with a mean age of 40.8±11 years. Their clinical features are summarized in Table 1. All subjects were non-obese, normotensive, normolipidemic, and euglycemic at fasting state. Carotid artery echocardiography showed no plaque lesions, aneurysmal changes or increase in pulsatility index (data not shown).

### Diminished flow-mediated vasodilatation after early repetitive examination

**Figure 1:**
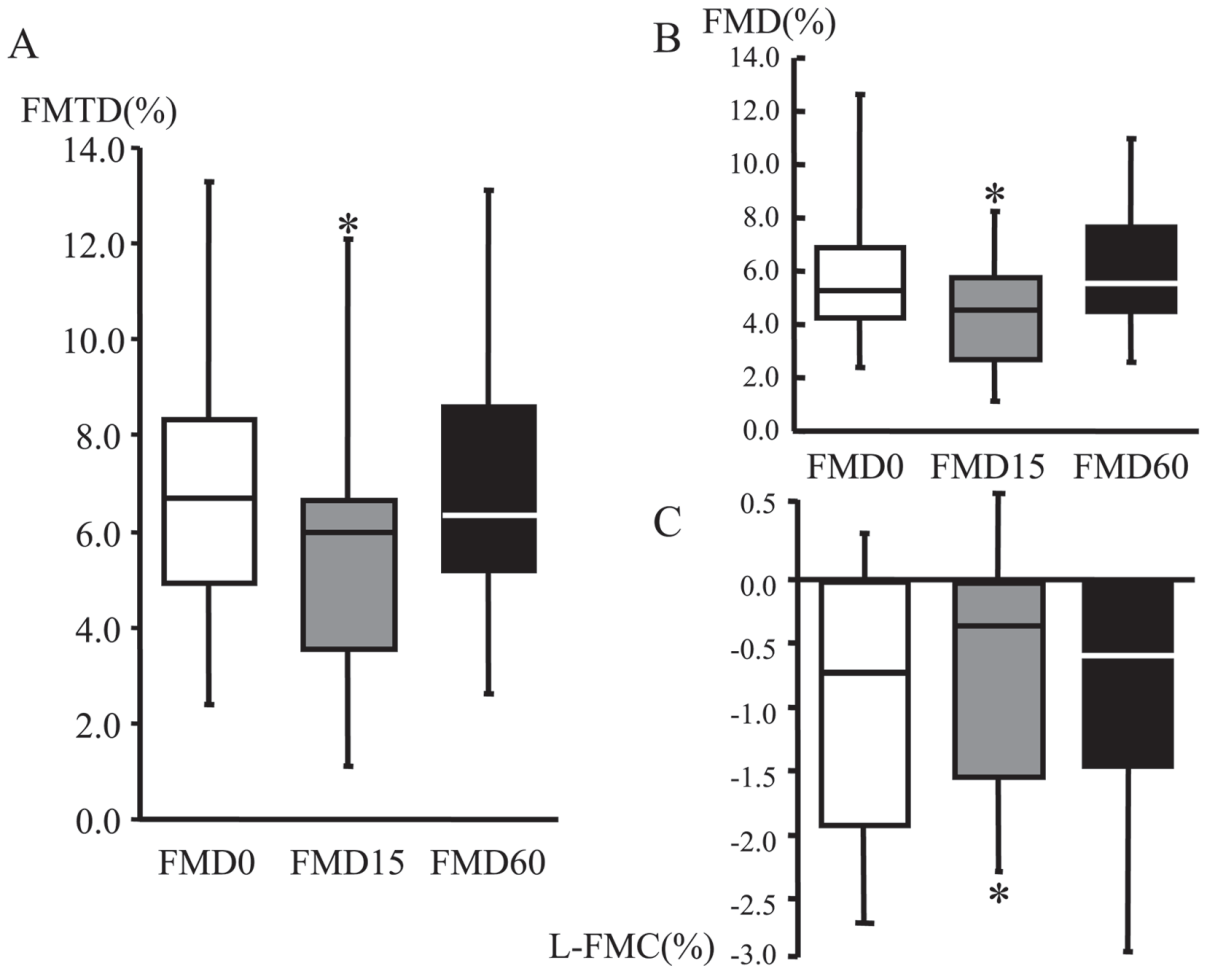
Serial changes in the physiological indices of flow-mediated vasodilatation. Flow-mediated total dilation (FMTD) (A), flow-mediated dilation (FMD) (B), and low–flow-mediated constriction (L-FMC) (C) values were obtained at 15 and 60 min intervals after the measurement. All indices were blunted at 15 min and showed complete recovery at 60 min. Data are mean ± SD. *p<0.05, compared with the respective baseline measurement.

Flow-mediated changes in radial artery diameter and blood flow velocity were sequentially observed at 15 and 60 min intervals after the examination and the indices were calculated. The baseline diameter was slightly but significantly increased at 15 min (p<0.01) and recovered at 60 min, but the maximum diameter unchanged (Table 2). Traditional FMD was within the normal range at 0 min [17]. The values of L-FMC, FMD and FMTD were significantly lower after 15 min interval (L-FMC, FMD and FMTD; 0 min and 15 min; −1.16±1.5 *vs* −0.94±1.3, 5.70±2.1 *vs* 4.27±2.0, and 6.60±2.4% *vs* 5.10±2.3, n = 30, p<0.05, respectively) (Fig. 1). The above indices returned to the baseline values after 60 min interval. The baseline and maximum flow velocity did not alter at 15 and 60 min (Table 2).

**Table 2:** Brachial artery characteristics.

	0	15	60	min	p Value
Baseline diameter (mm)	4.07±0.48	*4.11±0.48	4.09±0.45		P<0.01
Maximum diameter (mm)	4.30±0.49	4.29±0.47	4.33±0.49		NS
Baseline blood flow (cm/sec)	13.6±5.7	13.6±6.6	14.5±5.7		NS
Reactive blood flow (cm/sec)	48.4±25	56.3±26	54.8±26		NS

Values are mean ± SD.

p<0.01, compared with the respective baseline measurement.

### Recovery rate of FMDT correlates with L-FMC and CAVI

**Figure 2:**
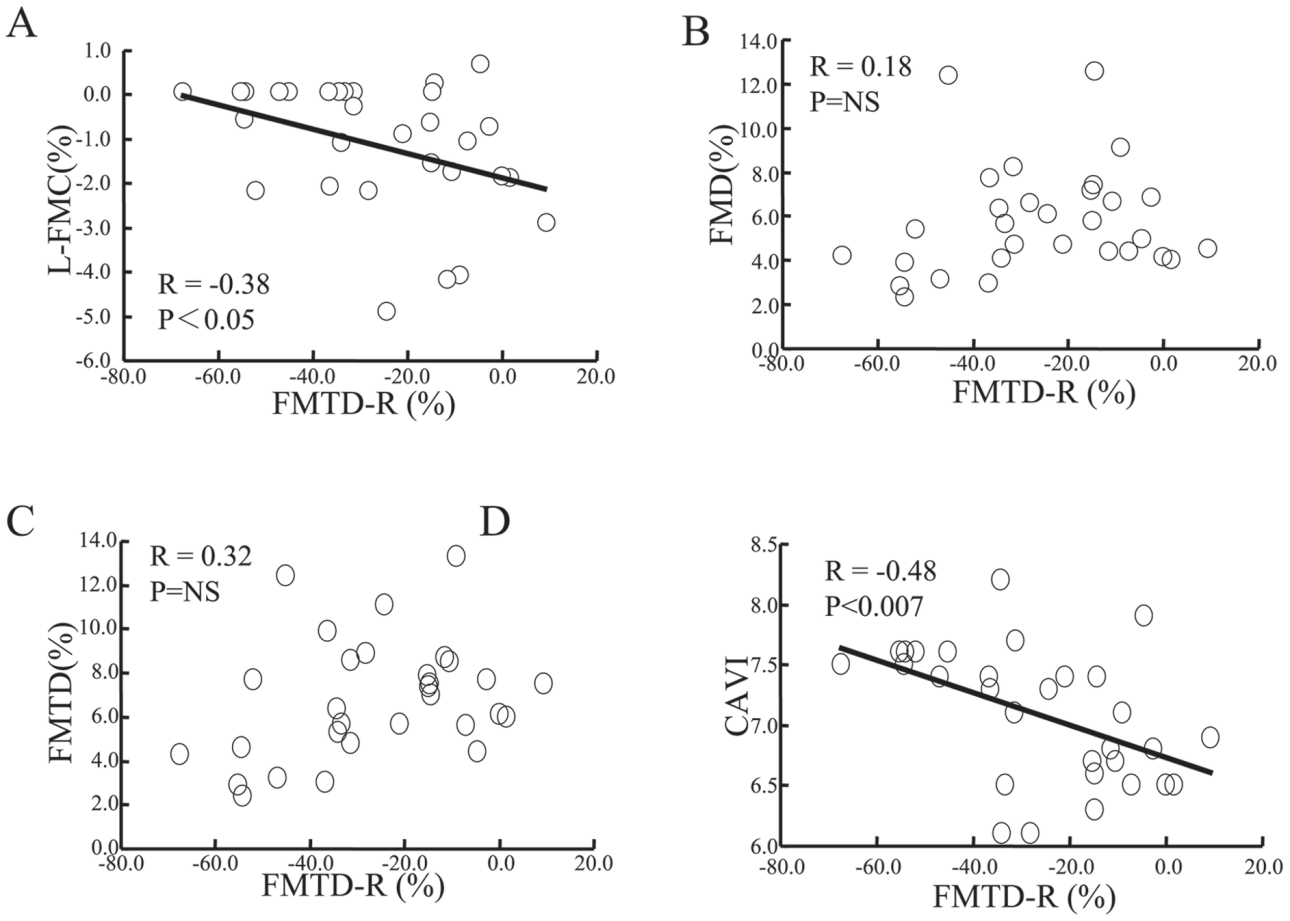
Correlation between FMTD-R and other FMD-related indices. Although a significant correlation between FMTD-R and L-FMC was confirmed (A), no relationship was found between FMTD-R and FMD (B) or FMTD (C). Notably, FMTD-R correlated significantly with CAVI (D).

Next, the relationships between FMTD-R and indices of flow-mediated vasodilatation were analyzed (Fig. 2). FMTD-R showed a moderate correlation with L-FMC (r = 0.38, p<0.05), but not with FMD or FMTD. FMTD-R also showed a moderate correlation with CAVI (r = −0.48, p<0.007).

### CAVI correlates with L-FMC

**Figure 3:**
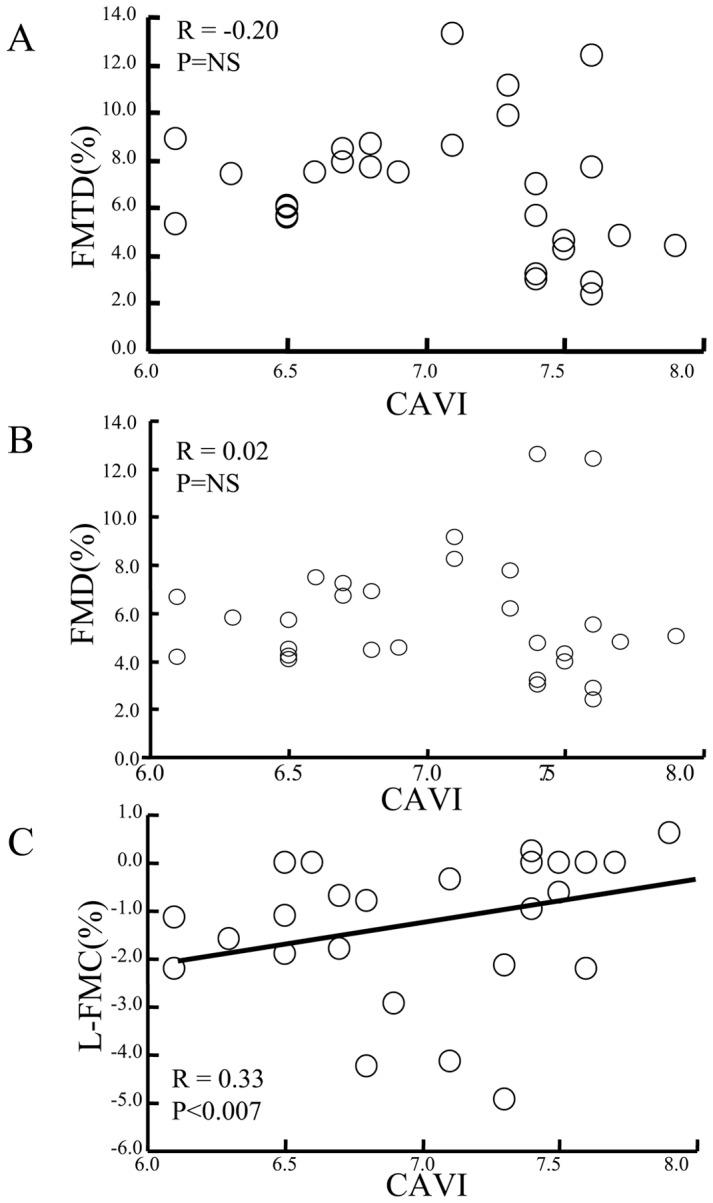
Correlation between CAVI and other FMD-related indices. Although a significant correlation between CAVI and L-FMC was confirmed (A), no relationship was found between CAVI and FMD (B) or FMTD (C).

We also analyzed the relationships between CAVI and indices of flow-mediated vasodilatation (Fig. 3). CAVI showed a weak correlation with L-FMC (r = 0.33, p<0.007) but no relation with FMD or FMTD.

### High CAVI in the poor FMD recovery group

**Figure 4:**
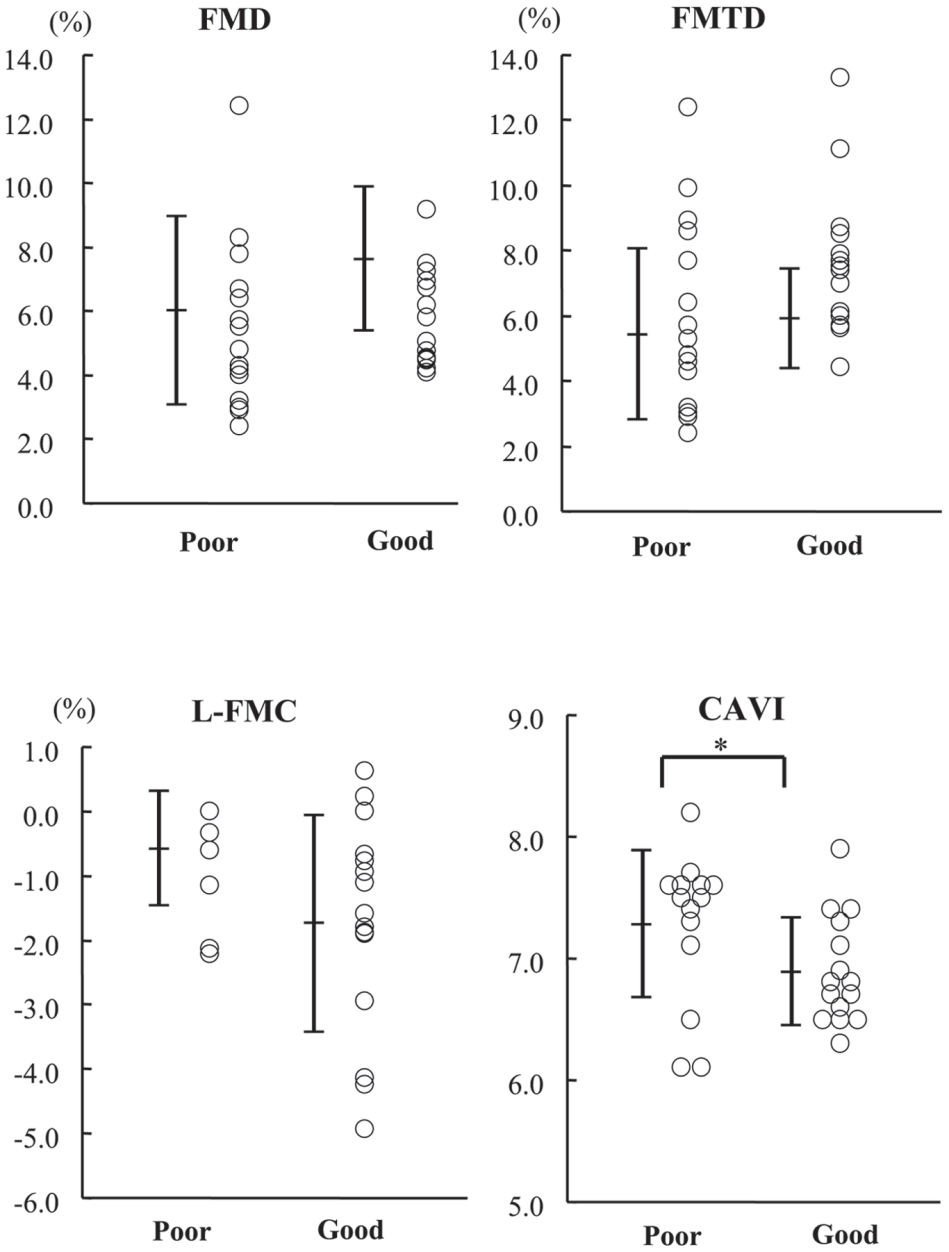
Values of FMD-related indices and CAVI in the healthy subjects divided into the poor and good recovery groups based on the median FMTD-R value (-26.2%). No significant difference was observed in FMD (A), FMTD (B), and L-FMC (C) values between the two groups. CAVI values were significantly higher in the poor recovery group.

Subjects were divided using the median FMTD-R value (−26.2%), into the good and poor recovery groups, and their FMD indices and CAVI were compared. The patient characteristics, with the exception of smoking status, were comparable between the two groups (Table 1). Furthermore, the FMD, L-FMC and FMTD values, and the diameter of the brachial artery were comparable between the good and poor recovery groups (Fig. 4). Interestingly, the poor recovery group included a higher percentage of current smokers (13.3% *vs* 66.7%; p<0.004) and the CAVI value was significantly higher than the good recovery group (6.89±0.44 *vs* 7.28±0.60; p<0.05).

### Reproducibility of FMTD-R and FMTD

The mean range of variation between the two sets of arterial diameter measurements using the software employed here was 0.015 mm. The respective mean ranges of interobserver variations for the measurement of FMTD-R and FMTD were 5.1% (0.01% to 14%) and 0.45% (0.018% to 2.9%) (n = 50 studies of arteries with a mean diameter of approximately 4.0 mm, n = 2 observers). The corresponding intraclass coefficients were 0.91 for FMTD-R and 0.96 for FMTD (p<0.01, respectively).

### Repeatability of FMTD-R and FMTD

**Figure 5:**
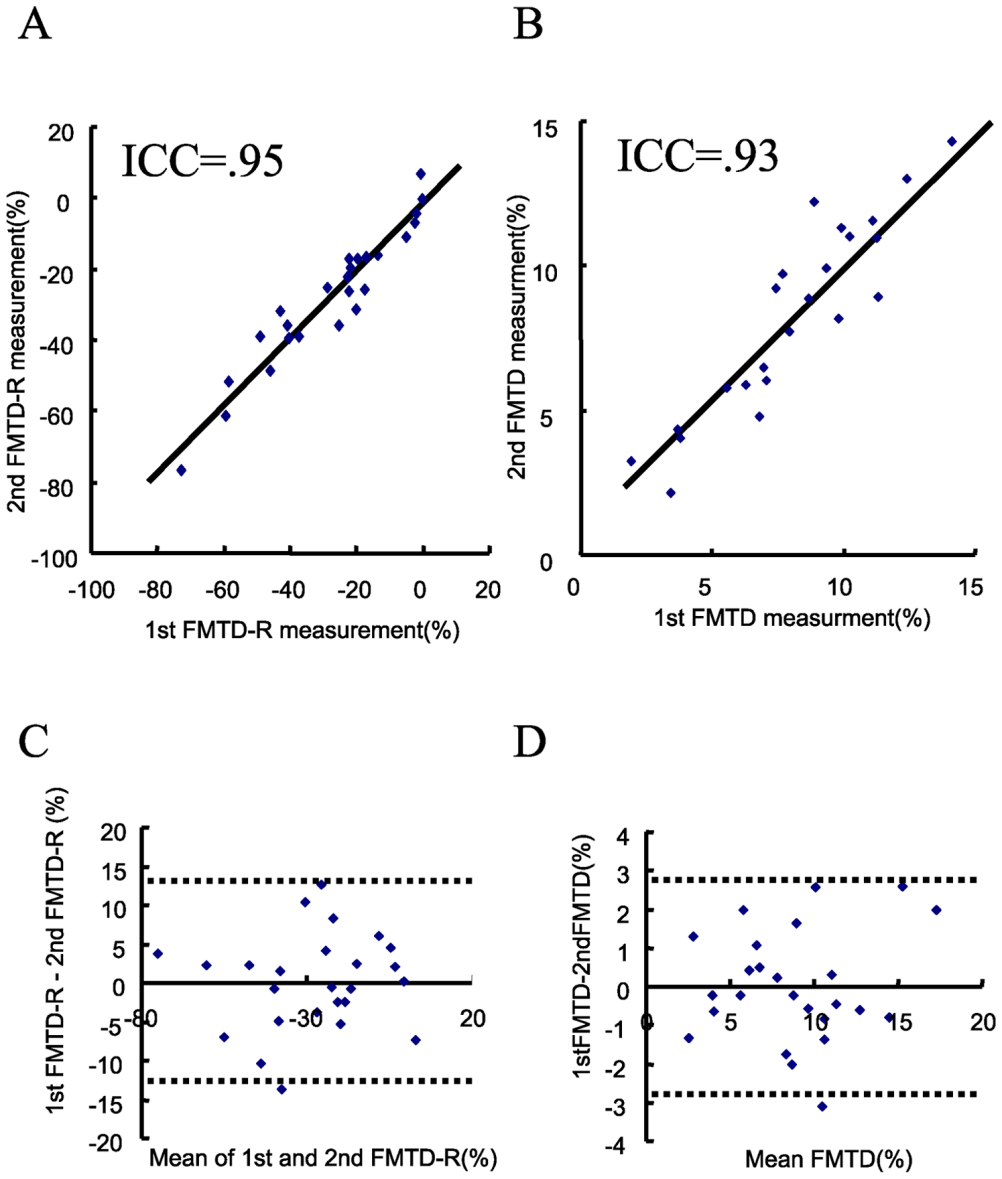
FMTD and FMTD-R measurements in consecutive FMD procedures in healthy volunteers. Correlation analysis for FMTD (A) and FMTD-R (B) (p<0.01, respectively.). Bland-Altman Plots for FMTD (C) and FMTD-R (D). The dotted lines represent 1.96 ± SD.

The intraclass correlation coefficients for repeated measurements of FMTD-R and FMTD, assessed in a subset of 25 patients, were 0.95 and 0.93 (Fig. 5A and B, p<0.01, respectively). The respective ranges of variation for FMTD-R and FMTD were 4.6% (0.01% to 11.5%) and 1.1% (0.01% to 2.6%). Figure 5C and D show the Bland and Altman plots for L-FMC and FMD measurements by the two independent observers. The coefficient of repeatability was 12.5% for FMTD-R and 2.86% for FMTD.

## Discussion

In the present study, we measured flow-mediated changes in radial artery diameter at 15 and 60 min intervals after the initial examination in healthy subjects. Notably, all indices of flow-mediated vasodilatation were blunted at 15 min (Fig. 1), but the decline showed complete recovery at 60 min (Fig. 1). The baseline diameter slightly increased at 15 min, which suggests delayed morphological restoration after the first flow-mediated dilatation, and was recovered at 60 min without changes in the maximum diameter and the baseline and reactive flow velocity (Table 2). The FMTD-R, which is assumed to reflect residual endothelial/vascular function in response to shear stress, correlated with L-FMC and CAVI (Figs. 2 and 3). CAVI also correlated with L-FMC (Fig. 2). The prevalence of smoking and values of CAVI were significantly higher in the FMTD-R poor recovery group than the good recovery group (Fig. 4). Measurements of both FMTD-R and FMTD showed good reproducibility with small coefficients of variation (Fig. 5). Considered collectively, the results of the present study suggest the possible involvement of FMTD-R in early vascular impairment in the healthy subjects.

### Appropriate interval for repetitive FMD measurements

Based on the international guidelines [13], measurements of FMD should be repeated after an interval up to 15 min. Our analysis showed blunted response to hyperemia and increased shear stress after the 15 min interval, suggesting inadequate recovery from the first dilation. We confirmed that measurements of FMTD-R and FMTD have excellent reproducibility and repeatability. The above finding is important because FMD measurement is often repeated in the clinical setting and research studies. Indeed, it has been documented that multiple FMD measurements can be validly performed when separated by ≥30 min intervals [15]. Although measurement of FMD is reproducible when performed within 5 min in healthy subjects, including aged individuals, males and females [18], further investigation is required to determine the most suitable interval for repetitive FMD measurement. Based on our tests, the interval of 15 min is inadequate for full recovery of FMD in middle-aged healthy males. Since a time delay for FMD after peak hyperemic flow is reportedly observed in healthy elderly with early endothelial impairment [19], blunted FMD after a short interval would be enhanced by the delayed response in the setting of endothelial dysfunction.

### Blunted FMD after short interval between measurements

Repeated FMD protocol within 15 min is reportedly regarded as the forearm ischemia-reperfusion model [20], [21]. Reactive oxygen species (ROS) were generated by the mitochondria, endothelial NO synthase (eNOS) uncoupling, xanthine oxidoreductase, and NADPH oxidase within vasculature in the setting of ischemia-reperfusion stress [22]. The bioavailability and production of NO were decreased by ROS production [23], resulting in attenuation of NO-dependent vasorelaxation such as FMD. Meanwhile, arterial stiffness depends on structural components such as extracellular matrix and arterial smooth muscle cell constriction. A number of locally derived and circulating factors, including NO, endothelium-derived hyperpolarizing factor, endothelin-1, and ROS, contribute to the short-term or functional regulation of arterial smooth muscle cells to regulate arterial stiffness and compliance [24]. Ischemia-reperfusion-mediated alteration in endothelium-derived factors would synergically lessen and blunt the vascular response. Reportedly, early vascular impairment in healthy elderly retards the vascular diameter changes in response to blood flow [25]. We also observed the delayed restoration of the vascular diameter at 15 min, which would be involved in a functional and transient arterial stiffness, resulting in a slight but significant increase in the baseline diameter. As the baseline diameter at 60 min and the maximum diameters at 15 min and 60 min unchanged, this would result in a significant decline in both FMD_15_ and L-FMC_15_, and low FMTD-R in accordance to respective equations [13] [6]. We observed the unchanged baseline and max flow velocity at all the time points. An increase in the baseline diameter with the same flow reduces a shear stress stimulus and FMD response [26].

In this study, we examined the relationship between FMTD-R and FMD-related indices. Interestingly FMTD-R did not correlate with FMD or FMTD, which are related to endothelium-related release of vasodilator mediators such as NO induced by high shear stress, and related to endothelial changes in response to increased shear stress. On the other hand, FMTD-R correlated with L-FMC, which is related to vasoconstrictor mediators such as endothelin-1 in the presence of low shear stress, and related to baseline endothelial function [27]. Thus, the blunted FMTD recovery after repetitive measurements can be involved in vasoconstriction rather than vasodilation. Previous studies demonstrated the association of various risk factors with impaired L-FMC [6], [8]. Indeed a higher prevalence of smoking is observed in the FMTD-R poor recovery group. Chronic cigarette smoking induces endothelial dysfunction and vasoconstriction with nicotine, reactive oxygen species and decreased NO bioavailability [28], [29]. Our results suggest that smoking, which is a major independent cardiovascular risk factor, contributes to the decrease in FMTD-R. Accordingly, FMTD-R as well as other FMD indices is involved also in latent cardiovascular risk.

### Aortic stiffness and FMD recovery after short interval between measurements

The present study showed for the first time that the aortic stiffness index, CAVI, is independent of FMD in normal subjects. This result is in agreement with the finding of a previous study that examined the relationship between PWV and FMD in control subjects [3]. On the other hand, our results suggested that CAVI correlated with FMTD-R. FMTD-R would be closely linked to CAVI because significantly high CAVI values were recorded in subjects of the poor FMTD-R group. CAVI is a novel index of systemic arterial stiffness, and is independent of blood pressure and linked to various cardiovascular risk factors, such as age, hypertension, diabetes mellitus, dyslipidemia, and considered a suitable marker of arteriosclerosis [5]. These results suggest that FMTD-R and CAVI are potentially useful markers of early arteriosclerosis. Our results also demonstrated a close relationship between CAVI and L-FMC. Interestingly, L-FMC is reportedly related to PWV, which is commonly used as an index of arterial stiffness, in healthy adults [30]. L-FMC is mediated through mechanisms independent of NO such as endothelin-1, cyclooxygenase products, and endothelial hyperpolarizing factor [6]. It would be suggested that CAVI and L-FMC are commonly regulated by NO-independent vasorelaxation and SMC function. These results imply the dependence of FMTD-R and L-FMC on vascular elasticity rather than flow-mediated vasodilation. Thus, vascular dysfunction in the early stages of atherosclerosis manifests as both endothelial impairment and arterial stiffness, in association with altered SMC function [4]. The use of the combination of these FMD-related indices; i.e., FMTD-R and CAVI, would provide a useful surrogate marker for early vascular impairment in a young healthy population.

### Limitations

The present study has several limitations. First, the automatic edge detection system, using continuous recordings of B-mode images and A-mode waves [14], does not make the entire method automated. The operator has to select areas of interest manually to achieve perfect visualization and parallelism of the artery walls. Using this system, however, we found excellent reproducibility and repeatability in measurements of FMTD and FMTD-R, though some degree of operator-driven variability is inevitable. Second, this study was solely conducted in a healthy young population. We did not measure FMD indices in diseased subjects with cardiovascular risk factors such as hypertension, diabetes and so on. We did not take account of the effect of any pathological process on FMD variability in our analysis. However, the aim of this pilot study was to determine the potential usefulness of this new method as a screening test for early vascular impairment in the healthy subjects. Further studies in a clinical or at-risk population suffering from endothelial dysfunction are warranted to further investigate the classification of early vascular impairment. Finally, we did not identify the mechanism of blunted flow-mediated indices 15 min after the first measurement. We assumed that FMTD-R is involved in the vasoconstriction mechanism based on its correlation with CAVI and L-FMC.

## Conclusions

In repetitive measurement of FMD, recovery is assumed after an adequate interval up to 15 min, but this has not been well investigated. We examined FMD recovery following sequential repetitive measurements, and hypothesized that recovery in a shorter period would be altered according to residual vascular function, and that impaired recovery correlates with early vascular dysfunction. The present pilot study demonstrated blunted recovery of FMD within 15 min in repeated measurements, suggesting the need for selection of a more adequate interval between measurements so as to avoid underestimation of FMD in subsequent measurements. Furthermore, our findings demonstrated the reproducibility of measurements of FMTD-R and FMTD, and that FMTD-R correlates with indices of arterial stiffness in the healthy subjects. Given the relationship between smoking and lower FMTD-R, one may hypothesize that the subjects with cardiovascular risk factors or diseases may have lower FMTD-R in accordance to increased arterial stiffness and vascular impairment.
